# Physical Inactivity Is Associated With Post-discharge Mortality and Re-hospitalization Risk Among Swedish Heart Failure Patients—The HARVEST-Malmö Study

**DOI:** 10.3389/fcvm.2022.843029

**Published:** 2022-02-21

**Authors:** Amir Zaghi, Hannes Holm, Johan Korduner, Anna Dieden, John Molvin, Erasmus Bachus, Amra Jujic, Martin Magnusson

**Affiliations:** ^1^Department of Clinical Sciences, Lund University, Malmö, Sweden; ^2^Department of Cardiology, Skane University Hospital, Lund University, Malmö, Sweden; ^3^Department of Internal Medicine, Skane University Hospital, Lund University, Malmö, Sweden; ^4^Department of Biomedical Science, Malmö University, Malmö, Sweden; ^5^Biofilms-Research Centre for Biointerfaces, Malmö University, Malmö, Sweden; ^6^Lund University Diabetes Centre, Lund University, Malmö, Sweden; ^7^Wallenberg Center for Molecular Medicine, Lund University, Lund, Sweden; ^8^Hypertension in Africa Research Team, North-West University, Potchefstroom, South Africa

**Keywords:** heart failure, cardiac rehabilitation, biomarkers, physical activity, risk factor

## Abstract

**Background:**

Several studies have examined the role of physical activity as a predictor of heart failure (HF) mortality and morbidity. Here, we aimed to evaluate the role of self-reported physical activity as an independent risk factor of post-discharge mortality and re-hospitalization in patients hospitalized for HF, as well as study the association between physical activity and 92 plasma proteins associated with cardiovascular disease (CVD).

**Methods:**

Four-hundred-and-thirty-four patients hospitalized for HF (mean age 75 years; 32% women) were screened for physical activity derived from questionnaires in the Swedish national public health survey. The median follow-up time to death and re-hospitalization was 835 (interquartile range, 390–1,432) and 157 (43–583) days, respectively. Associations between baseline reported physical activity, mortality and re-hospitalization risk were analyzed using multivariable Cox regression analysis. Plasma samples from 295 study participants were analyzed with a proximity extension assay consisting of 92 proteins. Associations between proteins and physical activity were explored using a false discovery rate of <5%, and significant associations were taken forward to multivariate analyses.

**Results:**

In the multivariate Cox regression model, physical inactivity, defined as physical activity time <1 h throughout the week was associated with increased risk of all-cause mortality (HR 1.71; CI95% 1.26–2.31; *p* = 5.9 × 10^−4^) as well as all-cause re-hospitalization (HR 1.27; CI95% 1.01–1.60; *p* = 0.038). Further, physical inactivity was associated with elevated plasma levels of Metalloproteinase inhibitor 4, Soluble interleukin 1 receptor-like 1, Elafin and Transferrin receptor protein 1, which are implicated in myocardial fibrosis, migration and apoptosis.

**Conclusions:**

Self-reported low weekly physical activity is associated with increased risk of mortality and re-hospitalization in patients hospitalized for HF independent of traditional risk factors. Furthermore, physical inactivity was associated with elevated levels of 4 proteins linked to cardiovascular disease.

## Key Messages

*What is already known about this subject?*

Several studies in the past have supported the role of physical activity as predictor of heart failure (HF) morbidity and mortality.

*What does this study add?*

Here, we arrived at results that support the role of self-reported physical inactivity as an independent risk factor of post-discharge mortality and hospitalization in patients hospitalized for heart failure, as well as finding an association between plasma levels of proteins associated with cardiovascular disease (CVD) and re-hospitalization and mortality in patients hospitalized for hospitalized for heart failure.

*How might this impact on clinical practice?*

This could potentially further support the prognostic role of physical inactivity prior to hospitalization for heart failure as a risk factor for post-discharge mortality and re-hospitalization in HF patients.

## Introduction

Heart failure (HF) is globally gaining in prevalence with an increasing burden on societies both in terms of quality of life and healthcare expenditures (1). While the focus over recent decades has been on pharmaceutical therapy, there is growing evidence suggesting that cardiac rehabilitation through physical exercise could reduce HF morbidity and mortality (2). In the 2021 guidelines of HF treatment from European Society of Cardiology (ESC), physical activity through cardiac rehabilitation centers is regarded as a Class I, Level A recommendation (3). However, only 10% of the eligible HF patients receive cardiac rehabilitation after hospitalization according to a data registry in a US setting (4). The inverse association between cardiorespiratory fitness and risk of cardiovascular disease as well as the value of cardiorespiratory fitness in predicting cardiovascular events, has been reported in several epidemiological studies (5). While studies on the benefits of exercise-based cardiac rehabilitation in patients with chronic HF [more specifically HF with reduced ejection fraction (HFrEF)] have shown only modest benefit on mortality and hospitalization at a population level (6), reduction in these endpoints appeared to be significant in the elderly and ischemic patients (7). In other words, the data on the survival benefit of exercise-based cardiac rehabilitation appears to be most significant in patients who are in a vulnerable phase of HF, e.g., patients discharged after an episode of decompensated HF. A notable prospective observational study examining the predictive role of physical activity on mortality in patients with severe HF (NYHA class IIIB) showed a significant inverse association between physical activity and mortality but the association with re-hospitalization was not studied (8). Recently, the use of proteomic serum analysis has emerged as a tool to identify highly predictive proteins of cardiovascular disease (9). Data from several studies have identified multiple proteins that are associated with HF as well as HF prognosis (10, 11). However, while there are data on the association between physical activity and plasma proteins that are associated with cardiovascular disease, they are currently limited in HF populations (12, 13). Furthermore, while the role of physical activity as a predictor of mortality and hospitalization has been previously studied, it has oftentimes not specifically studied patients hospitalized for HF. Hence, the aim of this prospective study is to evaluate the role of physical activity as an independent risk factor of post-discharge mortality and re-hospitalization in patients that have been hospitalized for HF and to study the association of plasma levels of proteins linked to inflammation/cardiovascular disease with physical activity in HF patients.

## Methods

### Study Population

The HeARt and Brain Failure inVESTigation study (HARVEST) is an ongoing, prospective study undertaken in patients hospitalized for the diagnosis of HF (ICD-10: I50-) in the city of Malmö, Sweden (14). The inclusion criterion for the HARVEST study is admission to the department of internal medicine or cardiology for treatment of newly diagnosed or exacerbated chronic HF after hospitalization for heart failure. The only exclusion criterion is the inability to deliver informed consent. In cases of severe cognitive impairment, the relatives are informed and asked for permission on patient's behalf. The study was approved by the Ethical Review Board at Lund University, Sweden and the study complies with the Declaration of Helsinki. A written informed consent was obtained from all participants or relatives as described above. Insights from the patients were welcomed but they were not directly involved in planning of the study design or conduct, or reporting, or dissemination plans of our research.

### Clinical Assessment

Upon hospitalization and subsequent admission to the clinical wards, study participants were examined with anthropometric measurements, and blood samples were drawn after overnight fast. Body mass index (BMI) was calculated as kilograms per square meter, and data regarding the study participants' medication were collected. Prevalence of diabetes was defined as either self-reported diagnosis of type 2 diabetes, or use of antidiabetic medication, or fasting plasma glucose >7 mmol/L. Smoking status was self-reported as yes or no, where never smokers were regarded as non-smokers, and previous and present-day smokers were defined as smokers. Trained nurses measured blood pressure (BP) using a validated automated BP monitor Boso Medicus (Bosch + Sohn GmbH u. Co. KG, Jungingen, Germany). The upper arm cuff of appropriate size was placed on the right side, and the arm was supported at heart level. Hypertension was defined as either systolic blood pressure ≥140 mmHg and/or diastolic blood pressure ≥90 mmHg. Atrial fibrillation (AF) was defined as presence of AF on an electrocardiogram at the time of hospitalization or history of AF according to patient's medical documentation. Self-reported education level was categorized as <9 or ≥9 study years. Prior cardiovascular disease was defined as prior stroke or prior myocardial infarction. The diagnosis of chronic obstructive pulmonary disorder (COPD) was retrieved from patients' medical journal.

### Laboratory Assays

Measurements of total N-terminal prohormone BNP (NT-proBNP), creatinine, and other biomarkers were carried out at the Department of Clinical Chemistry, Skåne University Hospital in Malmö, participating in a national standardization and quality control system (EQUALIS). Plasma creatinine was measured using an enzymatic colorimetric assay with an IDMS-traceable calibrator on the Hitachi Modular P analysis system (Roche, Basel, Switzerland). The total analytical imprecision was 3.0% (with a concentration of 60 μmol/L in control sample) and 1.4% (with a concentration of 578 μmol/L in control sample; normal reference range: 60–105 μmol/L for men and 50–90 μmol/L for women).

Fasting N-terminal pro-brain natriuretic peptide (NTproBNP) was analyzed using a sandwich assay based on ElectroChemiLuminiscence Immunoassay (Cobas, Roche Diagnostic, Basel, Switzerland) (15).

### Proteomic Profiling

Plasma levels of 92 CVD proteins were analyzed by the Proximity Extension Assay (PEA) technique using the Proseek Multiplex CVD III 96 × 96 reagents kit (Olink Bioscience, Uppsala, Sweden) (16). This technique uses two oligonucleotide-labeled specific antibodies to bind to each target protein, which allows the formation of a polymerase chain reaction sequence that can then be detected and quantified. The CVD III panel consists of 92 proteins, with established or proposed associations with metabolism, inflammation and CVD. All 92 proteins were above detectable limits in >15% of the samples. All data are presented as arbitrary units. Across all assays, the mean intra-assay and inter-assay variations were observed to be 8.1 and 11.4%, respectively. Further information about the assays is available on the Olink homepage (http://www.olink.com).

### Assessment of Physical Activity

The self-reported physical activity of the participants in this study was assessed through a standardized validated questionnaire based upon the public health agency of Sweden (17) (Supplementary Table 1). To define physical activity, a variable was computed based on the second question of the questionnaire since it clearly identifies the duration of physical activity and those who engage in medium intensity physical activity (i.e., engaging in physical activity that makes you warm or perspire such as fast walking, gardening, heavy housework, cycling, and swimming for at least 1 h per week). *Three* groups were established depending on the duration at which the participants engaged in physical activity per week: (1) up to 1 h, (2) 1–3 h, and (3) 3 h or more. In order to identify those who are physically inactive, an additional variable was computed based on the same question above, with physical inactivity being defined as engaging in <1 h of medium intensity physical activity per week.

### Echocardiography

Conventional transthoracic echocardiograms (TTE) were obtained using a Philips IE33 (Philips, Andover, MA, USA) with a 1–5 MHz transducer (S5-1), or with a GE Vingmed Vivid 7 Ultrasound (GE, Vingmed Ultrasound, Horten, Norway) with a 1–4 MHz transducer (M3S). All studies were performed by experienced sonographers. Cine loops were obtained from standard views (parasternal long axis, apical 4- and 2-chamber). Measurements were done offline using Xcelera 4.1.1 (Philips Medical Systems, Netherlands) according to the recommendations of the American Society of Echocardiography.

### Endpoints

Mortality was defined as death by any cause (total mortality) and was retrieved from the National Board of Health and Welfare's Cause of Death Register. Data regarding first of any re-hospitalizations were retrieved from electronical medical charts (Melior, Siemens Health Services, Solna, Sweden). All subjects were followed from study inclusion until December 31, 2020.

### Statistics

Variables are presented as means [standard deviation (SD)] and medians (25–75 interquartile range). Answers from the questionnaire were used to calculate independent variables that estimate the level of physical activity and entered in separate models depending on duration of physical activity.

#### Survival Analyses

Associations between baseline levels of self-reported physical activity of medium intensity, mortality and re-hospitalization risk were analyzed using multivariable Cox regression analysis. Proportional hazard assumption test was not violated. *Model 1* was adjusted for age and sex and *Model 2* was further adjusted for body mass index (BMI), systolic blood pressure at admission, prevalence of chronic obstructive pulmonary disease (COPD), prevalence of diabetes, prior HF, education level, brain natriuretic peptide, acute admission, current smoking and prevalence of atrial fibrillation as independent variables. In a separate analysis NYHA at admission was added to model 2, but the results did not change significantly. The time variable was calculated as follow-up time between screening and date of first hospitalization or death, respectively, or for censored cases, 31 December 2020. All-cause mortality and first of any post-discharge hospitalizations were chosen as the primary endpoints. These endpoints have been used in previous observational studies on the prognostic role of physical activity in HF patients, thereby facilitating the comparability of the results (6–8).

#### Associations Between Proteins Implicated in CVD and Physical Inactivity

Proteins in the CVD III panel are expressed as normalized protein expression (NPX) which is on a log2-scale. To analyse the associations of the 92 markers with regards to their association to physical inactivity, defined as engaging in physical activity up to 1 h vs. more than 1 h a week, unadjusted logistic regressions were carried out. Adjusted *p*-values were calculated using the Benjamini-Hochberg method (18). A false discovery rate (FDR) of <5% was applied for identification of markers to be taken forward to analyses in Model 1 (adjusted for age and sex). Associations that remained significant (*p* < 0.05) in *Model 1* were further adjusted according to *Model 2*. Group differences in continuous variables between study participants were compared using one-way ANOVA test, whereas categorical variables were compared using Pearson's Chi-square test. Non-parametric continuous variables, such as NTproBNP, were compared using Kruskal-Wallis H test (one-way ANOVA on ranks). All analyses were performed using SPSS Windows version 25.0 and R version 4.0.4, and a nominal *p*-value of < 0.05 was considered statistically significant.

## Results

### Patient Characteristics

Between March 2014 and December 2020, a total of 475 consecutive patients hospitalized for HF were included and underwent clinical examination. Forty-two patients had missing values on relevant co-variates and/or physical activity questionnaires, rendering a study population of 434 eligible participants with complete dataset. Of these 434 participants, a total of 295 patients, recruited consecutively from study start March 2014 until March 2018, had been analyzed with a proximity extension assay consisting of 92 proteins. Echocardiographic measurements were available in 305 study participants.

The study population had a mean age of 75 years, consisted predominantly of men (68%), 36% had diabetes and a high proportion of patients (88%) belonged to NYHA-class III-IV (Table 1). During the follow-up period (March 2014 through December 2020), a total of 195 (45%) patients died. The most frequent cause of death was heart failure (*n* = 58) followed by cardiac arrest (*n* = 14), cancer (*n* = 6), stroke (*n* = 4), and acute myocardial infarction (*n* = 1), the rest of the recorded deaths were due to non-cardiovascular causes (*n* = 63) or undefined (*n* = 49). A total of 333 (77%) patients were re-hospitalized, with the most common reason for re-hospitalization being heart failure (*n* = 132), arrhythmia (*n* = 23), acute myocardial infarction (*n* = 3), angina pectoris (*n* = 1) and cardiac arrest (*n* = 1). The rest of the recorded re-hospitalizations (*n* = 172) were due to a diversity of diagnoses and were defined as other in the database. A total of 196 patients (45%) reported physical activity more than 1 h a week. HF patients who engaged in physical activity <1 h a week were older, more likely to be female, had a higher proportion of NYHA class III-IV and lower systolic and diastolic blood pressure (Supplementary Table 2). HF patients who reported engaging in physical activity ≥3 h per week were more likely to be younger, have lower probability of NYHA class III-IV and prior HF. Also, the diastolic blood pressure was higher in participants who reported physical activity ≥3 h per week (Supplementary Table 3). Body mass index did not differ between any of the groups (Supplementary Tables 2, 3).

**Table 1:** Characteristics of study participants (*n* = 434) at baseline.

**Baseline characteristic**	***n*** **= 434**
Age [years; (±SD)]	75 (12)
Sex [female *n*; (%)]	140 (32)
NYHA-class III-IV [*n*; (%)]	419 (88)
Current smoking [*n*; (%)]	52 (12)
BMI [kg/m^2^; (±SD)]	28 (6)
SBP [mmHg; (±SD)]	138 (27)
DBP [mmHg; (±SD)]	80 (16)
Diabetes [*n*; (%)]	157 (36)
AF [*n*; (%)]	270 (62)
NTproBNP [median pmol/L; (interquartile range)]	4,321 (1,033–7,609)
GFR [median mL/min; (interquartile range)] *n* = 329 (5 missing)	45 (3–90)
COPD [*n*; (%)]	77 (18)
Education level > 9 years [*n*; (%)]	(1,033–7,609) (1,033–7,609) 228 (53)
Ejection fraction [%; (±SD)], *n* = 306 (128 missing)	39 (16)
HFrEF [*n*; (%)]	153 (50)
HFmrEF [*n*; (%)]	62 (20)
HFpEF [*n*; (%)]	91 (30)
Prior heart failure [*n*; (%)]	287 (66)
Acute admission [*n*; (%))]	328 (88)

*Values are means (±standard deviation), medians (interquartile range) or numbers (%). NYHA-class, New York heart association; BMI, Body mass index; SBP, Systolic blood pressure; DBP, Diastolic blood pressure; AF, Atrial fibrillation; NTproBNP, N terminal pro-atrial natriuretic peptide; COPD, chronic obstructive pulmonary disease; HFrEF, heart failure with reduced ejection fraction; HFmrEF, heart failure with midely reduced ejection fraction; HFpEF, heart failure with preserved ejection fraction*.

### Associations Between Physical Activity, Mortality, and Re-hospitalization Risk

**Figure 1:**
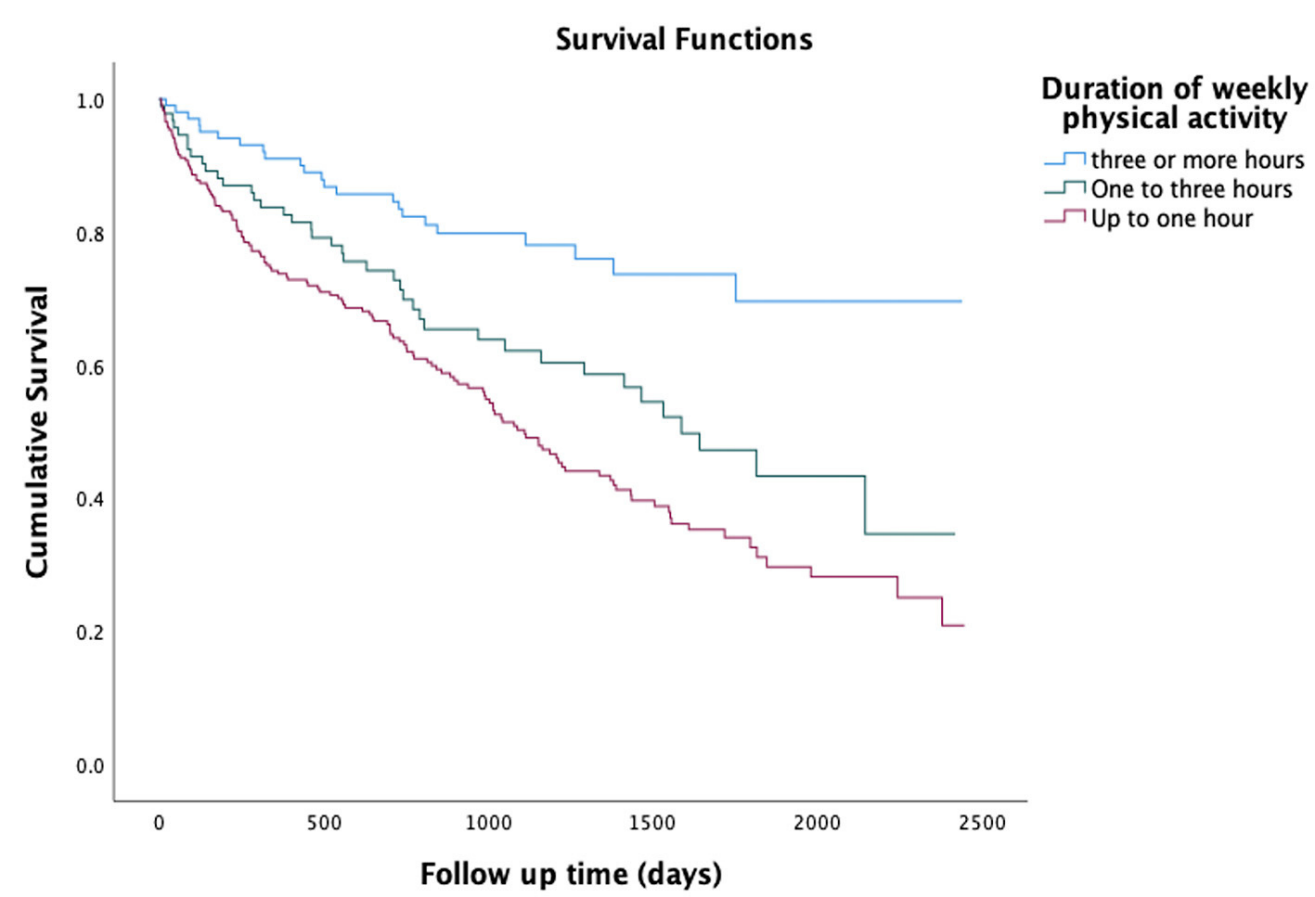
Kaplan-Meier curves for the risk of death amongst 434 participants in HARVEST stratified according to duration of weekly physical activity in the past 12 months prior hospitalization for HF.

In the fully adjusted Cox regression model, physical inactivity, i.e., engaging in <1 h of medium intensity physical activity per week, was associated with increased mortality (HR 1.71; CI95% 1.26–2.31; *p* = 5.9 × 10^−4^) over a follow-up time of 2,447 days, Table 2. Unadjusted Kaplan–Meier curves illustrating the risk of death in groups stratified according to duration of weekly physical activity (**<***1 h vs. 1–3 h vs*. ≥*3 h*) are shown in Figure 1. Physical inactivity was also associated with re-hospitalization risk in the fully adjusted Cox regression model (HR 1.27; CI95% 1.01–1.60; *p* = 0.038) (Table 2) over a follow-up time of 2,447 days. After adjustment for traditional risk factors, the Cox regression analysis on physical activity stratified into three groups (< *1 h vs. 1–3 h vs*. ≥*3 h*) also showed that participants with physical inactivity (<1 h) had increased risk of mortality (HR 2.34; CI95% 1.49–3.68; *p* = 2.4 × 10^−4^) and re-hospitalization (HR 1.42; CI95% 1.06–1.89; *p* = 0.019) compared to the reference group (physical activity ≥3 h), Table 3.

**Table 2:** Cox regression analysis displaying the association between physical inactivity (<1 h per week) and risk of mortality and re-hospitalization.

	**Mortality**		**Re-hospitalization**	
**Physical inactivity**	**HR (CI 95%)**	***p*** **-value**	**HR (CI 95%)**	***p*** **-value**
Model 1*	1.91 (1.42–2.59)	2.3 × 10^−5^	1.35 (1.08–1.69)	0.008
Model 2**	1.71 (1.26–2.31)	5.9 × 10^−4^	1.27 (1.01–1.60)	0.038

* *Model 1: Adjusted for sex, age*.

** *Model 2: Adjusted for sex, age, body mass index, systolic blood pressure at admission, prevalence of chronic obstructive pulmonary disease, prevalence of diabetes, prior heart failure, education, brain atrial natriuretic peptide, acute admission, current smoking and prevalence of atrial fibrillation*.

**Table 3:** Cox regression analysis displaying the association between duration of weekly physical activity and risk of re-hospitalization.

	**Mortality**		**Re-hospitalization**	
**Physical activity (<1 vs. ≥3h)**	**HR (CI 95%)**	***p*** **-value**	**HR (CI 95%)**	***p*** **-value**
Model 1*	2.59 (1.66–4.05)	2.8 × 10^−5^	1.51 (1.13–2.00)	0.005
Model 2**	2.34 (1.49–3.68)	2.4 × 10^−4^	1.42 (1.06–1.89)	0.019
**Physical activity (< 1 h vs. 1–3 h)**
Model 1*	1.48 (1.03–1.07)	0.029	1.24 (0.94–1.54)	0.121
Model 2**	1.43 (1.00–2.04)	0.052	1.22 (0.92–1.61)	0.161

* *Model 1: Adjusted for sex, age*.

** *Model 2: Adjusted for sex, age, body mass index, systolic blood pressure at admission, prevalence of chronic obstructive pulmonary disease, prevalence of diabetes, prior heart failure, education, brain atrial natriuretic peptide, acute admission, current smoking, and prevalence of atrial fibrillation*.

### Associations Between Physical Activity and Cardiovascular Biomarkers

A complete list of all proteins with their full names included in the analyses, is presented in the Supplementary Table 4. The association between proteins in the CVD III panel and physical inactivity both as unadjusted logistic regression analyses and adjusted *p*-values are presented in Supplementary Table 5. Proteins with an Benjamini-Hochberg adjusted *p*-value < 0.05 that remained significantly associated with medium intensity physical activity (< *1 vs*. ≥*1 h*) after adjustment for age and sex were further adjusted according to *Model 2* (Table 4), resulting in four proteins significantly associated with physical inactivity, namely Metalloproteinase inhibitor 4 (TIMP4), soluble interleukin 1 receptor-like 1 (ST2), Elafin (PI3) and Transferrin receptor protein 1 (TR).

**Table 4:** Logistic regression analysis examining the association of proteins predictive of cardiovascular disease to physical inactivity (up to 1 h vs. more than 1 h a week).

**Protein**	**Model 1**			**Model 2**		
	**OR**	**CI95%**	***p*** **-value**	**OR**	**CI95%**	***p*** **-value**
TIMP4	1.71	1.14–2.56	0.010	1.80	1.16–2.81	**0.009**
ST2	1.44	1.10–1.88	0.009	1.51	1.10–2.07	**0.011**
PI3	1.53	1.14–2.04	0.004	1.47	1.08–2.01	**0.015**
TR	1.47	1.08–1.99	0.013	1.48	1.07–2.05	**0.019**
TFF3	1.48	1.06–2.06	0.022	1.44	0.98–2.10	0.063
CD163	1.49	1.00–2.21	0.051	1.48	0.96–2.29	0.076
CSTB	1.39	1.03–1.89	0.032	1.31	0.93–1.83	0.119
Gal-3	1.72	1.05–2.81	0.030	1.65	0.98–2.77	0.058
FABP4	1.26	1.02–1.54	0.031	1.19	0.93–1.54	0.174
U-PAR	1.57	1.00–2.48	0.050	1.49	0.89–2.50	0.134
SPON1	1.54	0.88–2.71	0.132	1.54	0.84–2.81	0.162
IGFBP-2	1.37	0.96–1.97	0.086	1.43	0.94–2.18	0.094

*Logistic regressions for prevalence of physical inactivity adjusted for sex, age (Model 1) and age, sex, body mass index, New York Heart Association classification scale, prevalent diabetes, chronic obstructive pulmonary disorder, education, current smoking, brain atrial natriuretic peptide, and prevalence of atrial fibrillation (Model 2). Bolded values represent significant P-values in model 2*.

## Discussion

In this study, we showed that physical inactivity or the absence of regular weekly physical activity of medium intensity (≥1 h/week over the past 12 months prior to hospitalization for heart failure) is associated with increased risk for mortality and re-hospitalization, independent of traditional risk factors. In addition, increased plasma levels of four proteins related to CVD and inflammation were significantly associated with physical inactivity over the past 12 months prior to HF hospitalization.

These findings confirm the results of a recent observational study conducted on the role of physical activity as a predictor of mortality in HF patients (8). In 314 elderly patients (mean age 75 years) with HF in NYHA class IIIB, it was concluded that physical activity was inversely associated with mortality in elderly patients with advanced HF. This supports the reduction of mortality exerted by moderate physical activity in such patients. However, it should be noted that there is a limited number of observational studies investigating the role of physical activity as a predictor of post-discharge mortality and re-hospitalization in HF patients that have been hospitalized for heart failure. It should also be noted that a meta-analysis of randomized controlled trials on role of physical activity as a predictor of mortality and re-hospitalization has not shown promising results. One such meta-analysis is ExTraMATCH, which reported a reduction in all-cause mortality and reduction in the composite of mortality and hospital admission with exercise-based cardiac rehabilitation compared with no exercise control, but uncertainty arose as this study did not consider the cluster (or trial-level) nature of the data. Therefore, a second larger meta-analysis (ExTraMatch II) was recently conducted, not showing a significant association between physical activity and risk of mortality and re-hospitalization (7). The authors of this study, however, concluded that uncertainty around effect estimates precludes drawing definitive conclusion from the results. Still, there are randomized clinical trials (RCTs) that show significant reduction in all-cause mortality and re-hospitalization in HF patients that engage in physical activity. These include the HF-ACTION trial; a large US National Institute of Health funded trial with 2,331 HF patients recruited from 82 centers, showing that physical activity is associated with modest significant reduction of both all-cause mortality and re-hospitalization after adjustment for highly prognostic predictors (6). These contradicting results between observational studies, meta-analyses and RCTs on the role of physical activity as a predictor of mortality and re-hospitalization in HF patients reflect the complex nature of HF. One important distinction between observational studies and metanalyses on RCTs is that meta-regression analysis is highly prone to study level confounding and should be interpreted with caution. The conflicting results could also be attributed to the fact that the physical exercise in RCTs is more likely to be measured in the context of cardiac rehab, i.e., physical exercise utilized for its therapeutic potential, whereas in observational studies the physical activity more likely involves monitoring recreational or habitual physical activity. In other words, while both RCTs and observational studies could provide insight into the role of physical activity as an independent risk factor of mortality and re-hospitalization in HF patients, the context in which the physical activity is measured differs.

Both European Society of Cardiology (ESC) and American College of Cardiology (ACC) have used class I recommendation of regular physical activity as part of cardiac rehabilitation in HF patients (19, 20). These recommendations are in strong contrast to the general attitude of the medical community in the past as HF patients were generally recommended to maintain a less active lifestyle in order not to strain the heart. In the 1960s it was realized that even moderate amounts of physical exercise could lead to major health improvement in HF patients (21). It is of interest that this observation concurs with this study, as it appears that very moderate amounts of physical activity is sufficient to yield significant reduction in the risk of mortality and post-discharge hospitalization. Despite the mounting evidence in favor of cardiac rehab that involves physical activity in HF, it has not been widely adopted as part of the treatment regimen in HF patients and its adoption has often resulted in low adherence (22). This trend might change as an increasing number of guidelines aimed at cardiovascular health and rehabilitation are encouraging physical activity in both primary and secondary prevention of CVD (23). The assessment of physical activity in the context of health should take type of physical activity and dose (intensity, duration or frequency) into account (24). Self-reported physical activity has been shown to have poor reliability compared to objective measures, such as VO_2_ max and biomarkers of cardiorespiratory fitness, but it correlates well with health outcomes (24, 25).

There are several biomarkers that have been found to be associated with heart failure, and thereby used for risk stratification and prognosis. NTproBNP has been routinely used in the follow up of HF in clinical settings for a long time and it has long been considered the gold standard of biomarkers in establishing the diagnosis and prognosis of HF (26). Four of the plasma proteins [Metalloproteinase inhibitor 4 (TIMP-4), soluble interleukin 1 receptor-like 1 (ST2), Elafin (PI3), and Transferrin receptor protein 1 (TR) (27–29)] included in the proteomics panel analysis showed significant associations with physical inactivity, i.e., absence of medium intensity physical activity above 1 h a week. These proteins have been implicated in myocardial fibrosis, migration and apoptosis, which gives them a potential role in identifying risk factors that could contribute to pathologic heart remodeling and worsening HF prognosis.

ST2 is a validated prognostic marker in HF (30), but here, we could also show that higher ST2 levels were associated with physical inactivity. TIMP-4 has been shown to be highly expressed within the cardiovascular system, as well as in a number of other organs and tissues (31). A plasma multibiomarker panel including TIMP-4 predicted the presence of diastolic heart failure in subjects with left ventricular hypertrophy, implicating TIMP-4 in extracellular matrix fibrillar collagen homeostasis (32). A constant turnover of the extracellular matrix occurs during the progression from compensated hypertrophy to HF, where the altered balance between proteolysis/antiproteolysis results in fibrosis progression (33). Four days post-acute myocardial infarction, TIMP-4 was elevated and correlated positively with the occurrence of major adverse cardiac events, but in subjects with ischemic or idiopathic dilated cardiomyopathy, myocardial protein levels of TIMP-4 were reduced (34). Further, PI3 is an elastase-specific inhibitor produced by epithelial cells in response to macrophage infiltration, release of proteolytic enzymes, and disruption of epithelial integrity (35) with anti-inflammatory functions. Given that higher levels of elafin were associated with physical inactivity in our study, we believe that this association can be attributed to compensatory increment in elafin levels in physically inactive subjects. Furthermore, the similar compensatory increment might be possible for TR, which assists iron uptake. High levels of soluble transferrin receptor reflects depleted iron stores in bone marrow in patients with HF, and identifies those with a high 3-year mortality (36).

These findings compel the reflection of whether the predictive role of physical activity in HF mortality and re-hospitalization is associated with pathologic cardiac remodeling, and if physical activity could function as a preventive measure against pathologic cardiac remodeling and thereby potentially offer an improved prognosis in HF patients. This suggestion would concur with previous findings that have linked aerobic exercise with improved ejection fraction and the reversal of ventricular remodeling (37).

### Strengths and Limitations

The results obtained in this prospective study provide a compelling case for the role of physical activity as independent risk factor in post-discharge mortality and re-hospitalization in patients with advanced HF but the application of the results is limited since the physical activity of the cohort participants is only recorded once through a questionnaire instead of monitoring the physical activity of the cohort subjects continuously as they are followed over time. It is therefore not clear whether the physical activity habits of the study participants are consistent over the time during which they are being followed. It should also be noted that the survey might involve significant level of reporter bias, which would limit the applicability of the results. This risk could have been mitigated if the physical activity of the patients had been monitored with the help of step counter or other tools that can objectively measure physical activity. This can be considered for future studies in addition to randomization. It should also be noted that while the model adjusts for traditional prognostic factors like NT-proBNP, blood pressure, it does not adjust for other potentially important prognostic factors like baseline cardiorespiratory fitness prior to HF and as well as genetic preconditions, which could limit the conclusions based on the data. However, the HARVEST cohort is a relatively large and representative cohort that includes many relevant variables as well as rigorous endpoints. Blood samples were collected within 72 h from study inclusion after overnight fast. It is therefore possible that the initial medical treatment (including furosemide) for decompensated heart failure might have influenced the plasma levels of biomarkers included in the CVD III panel.

No causation can be drawn from the multivariable analysis aimed to find associations between physical inactivity and HF prognosis, nor in analysis directed to explore the relation between CVD biomarkers and physical inactivity. However, the fact that physical inactivity here can be linked to biomarkers that has previously been associated with HF prognosis, strengthens our hypothesis that physical inactivity is related to poor prognosis in HF.

Finally, in the current study, 128 participants had missing data for echocardiography. Hence, the left ventricular function has not been entered as a confounder in the survival analysis. However, ejection fraction as a measure of LV function did not differ between the groups of physical activity.

## Conclusions

Here we demonstrated that in patients hospitalized for HF, the absence of weekly medium intensity physical activity ≥1 h is associated with increased risk of post-discharge mortality and rehospitalization, independently of traditional risk factors. Also, 4 proteins implicated in cardiovascular disease were significantly associated with the absence of weekly medium intensity physical activity ≥1 h. This raises the question as to whether physical activity prior to hospitalization for acute heart failure can constitute an independent risk factor of post-discharge mortality and re-hospitalization in HF patients.

## Data Availability Statement

The datasets presented in this study are subject to ethical restrictions, but remain available upon reasonable request to the corresponding author.

## Ethics Statement

The studies involving human participants were reviewed and approved by Ethical Review Board at Lund University. The patients/participants provided their written informed consent to participate in this study.

## Author Contributions

The work has been mainly conducted by AZ (the content guarantor), with the assistance of HH, JK, AD, JM, EB, AJ, and MM. All authors contributed to the article and approved the submitted version.

## Funding

The HARVEST-Malmö Study was supported by grants from the Medical Faculty of Lund University (MM); Skane University Hospital (MM); the Crafoord Foundation (MM); the Ernhold Lundstroms Research Foundation (MM); the Region Skane (MM and AJ); the Hulda and Conrad Mossfelt Foundation (MM); the Southwest Skanes Diabetes Foundation (MM); the Kockska Foundation (MM); the Research Funds of Region Skåne (MM); the Swedish Heart and Lung Foundation (2018-0260 to MM); the Wallenberg Center for Molecular Medicine, Lund University (MM); and Lund University (AJ).

## Conflict of Interest

EB was employed by AstraZeneca since 2019. The remaining authors declare that the research was conducted in the absence of any commercial or financial relationships that could be construed as a potential conflict of interest.

## Publisher's Note

All claims expressed in this article are solely those of the authors and do not necessarily represent those of their affiliated organizations, or those of the publisher, the editors and the reviewers. Any product that may be evaluated in this article, or claim that may be made by its manufacturer, is not guaranteed or endorsed by the publisher.
