# Supplementary material for: Physical Inactivity Is Associated With Post-discharge Mortality and Re-hospitalization Risk Among Swedish Heart Failure Patients—The HARVEST-Malmö Study
Source: Front Cardiovasc Med. 2022 Feb 21;9:843029. doi: 10.3389/fcvm.2022.843029 (PMC8899472; doi:10.3389/fcvm.2022.843029)
Supplement: Supplementary file 1 [file Data_Sheet_1.docx]

**Supplementary tables**

**Supplementary table 1.** The self-reported physical activity of the participants based on questionnaire from the public health agency of Sweden

**1. How much physical movement and exertion have you had *in the last 12 months*?** *If your level of activity varies, e.g. between summer and winter, try to find an average. Please mark only one alternative!*

1. **Sedentary leisure time** *You mostly spend your free time with reading, TV, cinema or other sedentary pastimes. You walk, cycle or otherwise exercise less than 2 hours a week.*
2. **Moderate exercise in leisure time***. You walk, cycle or otherwise exercise at least 2 hours a week, usually without sweating. Include in this walking to and from work, other walking,
   ordinary gardening, fishing, table tennis, bowling.*
3. **Moderate, regular exercise in leisure time***. You exercise regularly 1-2 times a week for at least 30 minutes each time, running, swimming, tennis, badminton or other activity that makes you sweat.*
4. **Regular exercise and training***. You exercise by e.g. running, swimming, tennis, badminton, gymnastics or similar on average at least 3 times a week. This lasts for at least 30 minutes each time.*

**2. How much time do you spend in a normal week in moderately strenuous activities that make you warm?** *For example, walking fast, gardening, heavy housework, cycling, swimming. This may vary during the year, but try to give some kind of average. Choose one alternative.* 1.

1. 5 hours or more a week
2. More than 3 hours a week and less than 5
3. Between 1 and 3 hours a week
4. No more than one hour a week
5. Not at all

**3. Do you want to increase your physical activity?**

1. Yes, and I believe I will be able to do this myself
2. Yes, but I need support
3. No

**Supplementary Table 2.** Characteristics of study participants (n = 434) at baseline stratified according to physical activity amounting to up to one hour or more than one hour per week

| **Baseline characteristic** | Physical activity up to one hour per week  **n= 238** | Physical activity more than one hour per week  **n=196** | p-value |
| --- | --- | --- | --- |
| Age (years; (SD)) | 77 (11) | 72 (13) | 3.8x10^-4^ |
| Sex (female n;(%)) | 88 (37) | 52 (27) | 0.021 |
| NYHA-class III-IV (n; (%)) | 219 (92) | 163 (83) | 1.2x10^-4^ |
| Current smoking (n; (%)) | 30 (13) | 22 (11) | 0.795 |
| BMI (kg/m^2^; (SD)) | 28 (7) | 28 (5) | 0.459 |
| SBP (mmHg; (SD)) | 136 (25) | 141 (29) | 0.034 |
| DBP (mmHg; (SD)) | 78 (14) | 82 (18) | 0.049 |
| Diabetes (n; (%)) | 94 (40) | 63 (32) | 0.113 |
| AF (n; (%)) | 151 (63) | 119 (61) | 0.559 |
| COPD (n; (%)) | 49 (21) | 28 (14) | 0.231 |
| Education level ≥ 9 years (n; (%)) | 133 (56) | 95 (49) | 0.124 |
| Prior heart failure (n; (%)) | 170 (71) | 117 (60) | 0.058 |
| Acute admission (n; (%)) | 186 (78) | 142 (72) | 0.169 |
| Marital status |  |  | 0.664 |
| Married (n; (%)) | 27 (50) | 18 (39) |  |
| Single (n; (%)) | 7 (13) | 6 (13) |  |
| Divorced (n; (%)) | 5 (9) | 7 (15) |  |
| Widowed (n; (%)) | 15 (28) | 15 (33) |  |
| Ejection fraction (%; (± SD)) | 38 (16) | 39 (16) | 0.516 |
| HFrEF (n; (%)) | 78 (52) | 75 (48) | 0.493 |
| HFmrEF (n; (%)) | 29 (20) | 33 (21) | 0.777 |
| HFpEF (n; (%)) | 42 (28) | 48 (31) | 0.706 |
| NTproBNP (median pmol/L; (IQR)) | 4806 (1309-8303) | 3743 (479-7007) | 0.090 |
| GFR (median mL/min; (IQR)) | 49 (11-90) | 42 (3-90) | 0.002 |
| Beta blockade (n; (%)) | 176 (91) | 207 (86) | 0.226 |
| ACE inhibitors (n; (%)) | 105 (54) | 122 (51) | 0.562 |
| Loop diuretics (n; (%)) | 187 (97) | 231 (97) | 1.000 |
| Angiotensin receptor blockade (n; (%)) | 54 (28) | 66 (28) | 1.000 |

NYHA-class, New York heart association; BMI, Body mass index; SBP, Systolic blood pressure; DBP, Diastolic blood pressure; AF, Atrial fibrillation; COPD, Chronic obstructive pulmonary disease; NT-proBNP, N-terminal prohormone brain natriuretic peptide; GFR, glomerular filtration rate; ACE, Angiotensin converting enzyme

**Supplementary Table 3.** Characteristics of study participants (n = 434) at baseline stratified according to duration medium intensity physical activity

| **Baseline characteristic** | **Up to one hour**  **n= 238** | **One to three hours**  **n=93** | ***Three or more hours***  ***n=103*** | ***p-value*** |
| --- | --- | --- | --- | --- |
| Age (years; (SD)) | 77 (11) | 75 (12) | 70 (13) | 1.0x10^-4^ |
| Sex (female n;(%)) | 88 (37) | 27 (29) | 25 (24) | 0.053 |
| NYHA-class III-IV (n; (%)) | 219 (92) | 79 (85) | 88 (85) | 0.001 |
| Current smoking (n; (%)) | 30 (13) | 8 (9) | 14 (14) | 0.510 |
| BMI (kg/m^2^; (SD)) | 28 (7) | 28 (5) | 28 (5) | 0.761 |
| SBP (mmHg; (SD)) | 136 (25) | 139 (29) | 144 (30) | 0.053 |
| DBP (mmHg; (SD)) | 78 (14) | 80 (17) | 84 (19) | 5.0x10^-3^ |
| Diabetes (n; (%)) | 94 (39) | 36 (39) | 27 (26) | 0.054 |
| AF (n; (%)) | 151 (63) | 64 (69) | 55 (53) | 0.071 |
| COPD (n; (%)) | 49 (21) | 14 (15) | 14 (14) | 0.407 |
| Education level ≥ 9 years (n; (%)) | 133 (56) | 49 (53) | 46 (45) | 0.163 |
| Prior heart failure (n; (%)) | 170 (71) | 62 (67) | 55 (53) | 0.021 |
| Acute admission (n; (%)) | 186 (78) | 70 (75) | 72 (70) | 0.265 |
| NTproBNP (median pmol/L; (IQR)) | 4806 (1309-8303) | 3820 (98-7542) | 3538 (269-6808) | 0.076 |

Values are means (± standard deviation), medians (interquartile range) or numbers (%). NYHA-class, New York heart association; BMI, Body mass index; SBP, Systolic blood pressure; DBP, Diastolic blood pressure; AF, Atrial fibrillation; COPD, Chronic obstructive pulmonary disease; NTproBNP**,** N-terminal pro brain natriuretic peptide.

# Supplementary table 4. List of all 92 proteins included in analyses

| Aminopeptidase N (AP-N) |
| --- |
| Azurocidin (AZU1) |
| Bleomycin hydrolase (BLM hydrolase) |
| C-C motif chemokine 15 (CCL15) |
| C-C motif chemokine 16 (CCL16) |
| C-C motif chemokine 24 (CCL24) |
| C-X-C motif chemokine 16 (CXCL16) |
| Cadherin-5 (CDH5) |
| Carboxypeptidase A1 (CPA1) |
| Carboxypeptidase B (CPB1) |
| Caspase-3 (CASP-3) |
| Cathepsin D (CTSD) |
| Cathepsin Z (CTSZ) |
| CD166 antigen (ALCAM) |
| Chitinase-3-like protein 1 (CHI3L1) |
| Chitotriosidase-1 (CHIT1) |
| Collagen alpha-1(I) chain (COL1A1) |
| Complement component C1q receptor (CD93) |
| Contactin-1 (CNTN1) |
| Cystatin-B (CSTB) |
| E-selectin (SELE) |
| Elafin (PI3) |
| Ephrin type-B receptor 4 (EPHB4) |
| Epidermal growth factor receptor (EGFR) |
| Epithelial cell adhesion molecule (Ep-CAM) |
| Fatty acid-binding protein, adipocyte (FABP4) |
| Galectin-3 (Gal-3) |
| Galectin-4 (Gal-4) |
| Granulins (GRN) |
| Growth/differentiation factor 15 (GDF-15) |
| Insulin-like growth factor-binding protein 1 (IGFBP-1) |
| Insulin-like growth factor-binding protein 2 (IGFBP-2) |
| Insulin-like growth factor-binding protein 7 (IGFBP-7) |
| Integrin beta-2 (ITGB2) |
| Intercellular adhesion molecule 2 (ICAM-2) |
| Interleukin-1 receptor type 1 (IL-1RT1) |
| Interleukin-1 receptor type 2 (IL-1RT2) |
| Interleukin-17 receptor A (IL-17RA) |
| Interleukin-18-binding protein (IL-18BP) |
| Interleukin-2 receptor subunit alpha (IL2-RA) |
| Interleukin-6 receptor subunit alpha (IL-6RA) |
| Junctional adhesion molecule A (JAM-A) |
| Kallikrein-6 (KLK6) |
| Low-density lipoprotein receptor (LDL receptor) |
| Lymphotoxin-beta receptor (LTBR) |
| Matrix extracellular phosphoglycoprotein (MEPE) |
| Matrix metalloproteinase-2 (MMP-2) |
| Matrix metalloproteinase-3 (MMP-3) |
| Matrix metalloproteinase-9 (MMP-9) |
| Metalloproteinase inhibitor 4 (TIMP4) |
| Monocyte chemotactic protein 1 (MCP-1) |
| Myeloblastin (PRTN3) |
| Myeloperoxidase (MPO) |
| Myoglobin (MB) |
| N-terminal prohormone brain natriuretic peptide (NT-proBNP) |
| Neurogenic locus notch homolog protein 3 (Notch 3) |
| Osteopontin (OPN) |
| Osteoprotegerin (OPG) |
| P-selectin (SELP) |
| Paraoxonase (PON3) |
| Peptidoglycan recognition protein 1 (PGLYRP1) |
| Perlecan (PLC) |
| Plasminogen activator inhibitor 1 (PAI) |
| Platelet endothelial cell adhesion molecule (PECAM-1) |
| Platelet glycoprotein VI (GP6) |
| Platelet-derived growth factor subunit A (PDGF subunit A) |
| Proprotein convertase subtilisin/kexin type 9 (PCSK9) |
| Protein delta homolog 1 (DLK-1) |
| Pulmonary surfactant-associated protein D (PSP-D) |
| Resistin (RETN) |
| Retinoic acid receptor responder protein 2 (RARRES2) |
| Scavenger receptor cysteine-rich type 1 protein M130 (CD163) |
| Secretoglobin family 3A member 2 (SCGB3A2) |
| Spondin-1 (SPON1) |
| ST2 protein (ST2) |
| Tartrate-resistant acid phosphatase type 5 (TR-AP) |
| Tissue factor pathway inhibitor (TFPI) |
| Tissue-type plasminogen activator (t-PA) |
| Transferrin receptor protein 1 (TR) |
| Trefoil factor 3 (TFF3) |
| Trem-like transcript 2 protein (TLT-2) |
| Tumor necrosis factor ligand superfamily member 13B (TNFSF13B) |
| Tumor necrosis factor receptor 1 (TNF-R1) |
| Tumor necrosis factor receptor 2 (TNF-R2) |
| Tumor necrosis factor receptor superfamily member 10C (TNFRSF10C) |
| Tumor necrosis factor receptor superfamily member 14 (TNFRSF14) |
| Tumor necrosis factor receptor superfamily member 6 (FAS) |
| Tyrosine-protein kinase receptor UFO (AXL) |
| Tyrosine-protein phosphatase non-receptor type substrate 1 (SHPS-1) |
| Urokinase plasminogen activator surface receptor (U-PAR) |
| Urokinase-type plasminogen activator (uPA) |
| von Willebrand factor (vWF) |

**Supplementary table 5.** Unadjusted associations between proteins and sedentary lifestyle with Benjamini-Hochberg multiple testing correction (adjusted p-values; false discovery rate 0.05)

| Protein | OR | CI95% | | p-value | Adjusted p-value |
| --- | --- | --- | --- | --- | --- |
| TFF3 | 1.66 | 1.23 | 2.24 | 0.001 | 0.018 |
| CSTB | 1.57 | 1.19 | 2.07 | 0.001 | 0.018 |
| TIMP4 | 1.83 | 1.28 | 2.62 | 0.001 | 0.018 |
| PI3 | 1.53 | 1.19 | 1.98 | 0.001 | 0.018 |
| ST2 | 1.53 | 1.18 | 1.99 | 0.001 | 0.018 |
| FABP4 | 1.37 | 1.12 | 1.67 | 0.002 | 0.023 |
| U-PAR | 1.94 | 1.27 | 3.00 | 0.002 | 0.023 |
| Gal3 | 2.07 | 1.31 | 3.29 | 0.002 | 0.023 |
| CD163 | 1.78 | 1.21 | 2.62 | 0.003 | 0.031 |
| TR | 1.49 | 1.13 | 1.98 | 0.005 | 0.042 |
| IGFBP-2 | 1.59 | 1.51 | 2.19 | 0.005 | 0.042 |
| SPON1 | 2.14 | 1.24 | 3.68 | 0.006 | 0.046 |
| TNF-R2 | 1.51 | 1.11 | 2.06 | 0.009 | 0.059 |
| PLC | 1.74 | 1.15 | 2.63 | 0.009 | 0.059 |
| MMP-3 | 1.36 | 1.08 | 1.72 | 0.010 | 0.061 |
| TNFRSF10C | 1.68 | 1.12 | 2.51 | 0.012 | 0.069 |
| Gal-4 | 1.55 | 1.09 | 2.21 | 0.015 | 0.077 |
| TNF-R1 | 1.50 | 1.08 | 2.09 | 0.015 | 0.077 |
| CCL24 | 1.32 | 1.05 | 1.66 | 0.017 | 0.082 |
| RETN | 1.48 | 1.06 | 2.07 | 0.020 | 0.092 |
| CHIT1 | 1.21 | 1.03 | 1.43 | 0.024 | 0.105 |
| Notch3 | 1.42 | 0.93 | 2.17 | 0.105 | 0.140 |
| TIMP4 | 2.13 | 1.47 | 3.09 | 0.000 | 0.001 |
| CNTN1 | 1.16 | 0.68 | 1.99 | 0.583 | 0..638 |
| CDH5 | 1.43 | 0.80 | 2.56 | 0.233 | 0.278 |
| TLT2 | 1.31 | 0.89 | 1.94 | 0.171 | 0.215 |
| FABP4 | 1.50 | 1.22 | 1.84 | 0.000 | 0.001 |
| TFPI | 1.61 | 0.93 | 2.82 | 0.092 | 0.124 |
| PAI | 1.31 | 0.98 | 1.76 | 0.074 | 0.106 |
| CCL24 | 1.32 | 1.05 | 1.67 | 0.017 | 0.041 |
| TR | 1.46 | 1.09 | 1.94 | 0.010 | 0.030 |
| TNFRSF10C | 1.66 | 1.10 | 2.50 | 0.017 | 0.041 |
| GDF15 | 1.47 | 1.13 | 1.92 | 0.004 | 0.016 |
| SELE | 1.44 | 1.00 | 2.08 | 0.053 | 0.085 |
| AZU1 | 1.67 | 1.19 | 2.35 | 0.003 | 0.013 |
| DLK1 | 1.37 | 1.02 | 1.83 | 0.034 | 0.061 |
| SPON1 | 1.88 | 1.09 | 3.24 | 0.023 | 0.051 |
| MPO | 1.70 | 1.05 | 2.75 | 0.032 | 0.058 |
| CXCL16 | 2.02 | 1.17 | 3.49 | 0.012 | 0.033 |
| IL6RA | 1.85 | 1.11 | 3.07 | 0.017 | 0.041 |
| RETN | 1.59 | 1.13 | 2.24 | 0.007 | 0.025 |
| IGFBP1 | 1.26 | 1.02 | 1.55 | 0.031 | 0.058 |
| CHIT1 | 1.28 | 1.07 | 1.54 | 0.007 | 0.025 |
| TRAP | 1.53 | 0.95 | 2.44 | 0.078 | 0.110 |
| GP6 | 1.52 | 1.07 | 2.16 | 0.018 | 0.042 |
| PSPD | 1.01 | 0.76 | 1.34 | 0.944 | 0.944 |
| PI3 | 1.87 | 1.42 | 2.45 | 0.000 | 0.000 |
| EpCAM | 1.38 | 1.09 | 1.76 | 0.009 | 0.028 |
| APN | 1.09 | 0.72 | 1.68 | 0.678 | 0.733 |
| AXL | 1.71 | 1.04 | 2.82 | 0.035 | 0.061 |
| IL1RT1 | 1.83 | 1.10 | 3.05 | 0.021 | 0.048 |
| MMP2 | 1.15 | 0.78 | 1.69 | 0.492 | 0.545 |
| FAS | 1.19 | 0.79 | 1.78 | 0.405 | 0.454 |
| MB | 1.57 | 1.22 | 2.03 | 0.000 | 0.003 |
| TNFSF13B | 1.29 | 0.89 | 1.86 | 0.176 | 0.218 |
| PRTN3 | 1.88 | 1.30 | 2.71 | 0.001 | 0.006 |
| PCSK9 | 1.39 | 0.82 | 2.37 | 0.226 | 0.275 |
| UPAR | 2.69 | 1.70 | 4.27 | 0.000 | 0.001 |
| OPN | 1.43 | 1.05 | 1.96 | 0.024 | 0.052 |
| CTSD | 1.49 | 0.97 | 2.28 | 0.070 | 0.105 |
| PGLYRP1 | 2.03 | 1.37 | 3.01 | 0.000 | 0.003 |
| CPA1 | 0.97 | 0.79 | 1.19 | 0.774 | 0.818 |
| JAMA | 1.30 | 1.00 | 1.69 | 0.054 | 0.085 |
| Gal4 | 1.88 | 1.28 | 2.76 | 0.001 | 0.006 |
| IL1RT2 | 1.25 | 0.75 | 2.11 | 0.395 | 0.448 |
| SHPS1 | 1.61 | 1.04 | 2.49 | 0.031 | 0.058 |
| CCL15 | 1.61 | 1.11 | 2.33 | 0.013 | 0.035 |
| CASP3 | 1.35 | 1.04 | 1.75 | 0.026 | 0.053 |
| uPA | 1.26 | 0.75 | 2.11 | 0.390 | 0.448 |
| CPB1 | 0.98 | 0.77 | 1.24 | 0.856 | 0.875 |
| tPA | 1.28 | 0.98 | 1.69 | 0.073 | 0.106 |
| SCGB3A2 | 1.34 | 1.03 | 1.76 | 0.031 | 0.058 |
| EGFR | 1.13 | 0.52 | 2.47 | 0.754 | 0.806 |
| IGFBP7 | 1.38 | 1.01 | 1.89 | 0.046 | 0.076 |
| CD93 | 1.30 | 0.80 | 2.12 | 0.293 | 0.341 |
| IL18BP | 1.76 | 1.16 | 2.69 | 0.009 | 0.028 |
| COL1A1 | 0.97 | 0.63 | 1.49 | 0.870 | 0.879 |
| PON3 | 1.03 | 0.74 | 1.44 | 0.854 | 0.875 |
| CTSZ | 1.98 | 1.16 | 3.35 | 0.012 | 0.033 |
| MMP3 | 1.46 | 1.15 | 1.85 | 0.002 | 0.009 |
| RARRES2 | 1.95 | 1.05 | 3.62 | 0.036 | 0.062 |
| ICAM2 | 1.72 | 1.07 | 2.76 | 0.025 | 0.053 |
| KLK6 | 1.62 | 0.98 | 2.70 | 0.062 | 0.095 |
| PDGFsubunitA | 1.30 | 0.97 | 1.74 | 0.080 | 0.111 |
| TNFR1 | 1.98 | 1.39 | 2.81 | 0.000 | 0.001 |
| IGFBP2 | 1.70 | 1.21 | 2.38 | 0.002 | 0.009 |
| vWF | 1.35 | 1.04 | 1.77 | 0.026 | 0.053 |
| PECAM1 | 1.38 | 0.99 | 1.92 | 0.060 | 0.093 |
| MEPE | 1.29 | 0.92 | 1.79 | 0.140 | 0.181 |
| CCL16 | 1.22 | 0.88 | 1.69 | 0.243 | 0.286 |
| CHI3L1 | 1.36 | 1.10 | 1.68 | 0.004 | 0.016 |
| ST2 | 1.81 | 1.37 | 2.38 | 0.000 | 0.001 |
